# Syntheses and structures of spontaneously resolved (2*S*)-2-phenyl-3-(thia­zol-2-yl)-2,3,5,6-tetra­hydro-4*H*-1,3-thia­zin-4-one and racemic 2-(furan-2-yl)-3-phenyl-2,3,5,6-tetra­hydro-4*H*-1,3-thia­zin-4-one

**DOI:** 10.1107/S2056989025009193

**Published:** 2025-10-24

**Authors:** Hemant P. Yennawar, Tapas K. Mal, Mark A. Olsen, Anthony F. Lagalante, Aloura D. Gavalis, Isabella G. Frederick, Evelyn M. Louca, Lee J. Silverberg

**Affiliations:** a Department of Biochemistry and Molecular Biology The Pennsylvania State University University Park PA 16802 USA; b Department of Chemistry The Pennsylvania State University University Park PA 16802 USA; c Mendel Science Center Villanova University, 800 Lancaster Avenue Villanova PA 19085 USA; d Pennsylvania State University, Schuylkill Campus, 200 University Drive, Schuylkill Haven, PA 17972, USA; University of Aberdeen, United Kingdom

**Keywords:** crystal structure, spontaneous resolution, half-chair, thia­zine ring pucker

## Abstract

The title compounds, one a thia­zole derivative: (2*S*)-2-phenyl-3-(thia­zol-2-yl)-2,3,5,6-tetra­hydro-4*H*-1,3-thia­zin-4-one, C_13_H_12_N_2_OS_2_, and the second a furan derivative: (*rac*)-2-(furan-2-yl)-3-phenyl-2,3,5,6-tetra­hydro-4*H*-1,3-thia­zin-4-one, C_14_H_13_NO_2_S, crystallize in space-groups *P*2_1_2_1_2_1_ and *P*2_1_/*c*, respectively, with a single mol­ecule in their asymmetric units. The crystal of the thia­zole derivative chosen for data collection was found to consist of the *S* enanti­omer [Flack parameter 0.013 (9)]. The crystal of the furan derivative in the centrosymmetric space group is a racemic mixture. The puckering of the thia­zine ring in both the structures is a half-chair. The extended structure of the thia­zole derivative shows two weak C—H⋯O type inter­actions, but no aromatic ring inter­actions. In the structure of the furan derivative, an extensive and continuous network of C—H⋯O hydrogen bonds between the furan and the substituted thia­zine ring, and also between symmetry-related furan rings, results in a continuous amphiphilic layer lying parallel to the (100) plane. Adjacent to this plane is the layer of hydro­phobic phenyl rings. Thus, the extended structure comprises alternating layers of amphiphilic and hydro­phobic regions, stacked in the *a*-axis direction. A C—H⋯O inter­action between the phenyl ring and the thia­zin-4-one moiety and the π–π stacking of the phenyl rings between pairs of symmetry-related mol­ecules further consolidates the extended structure.

## Abstract 2

One of the title compounds shows spontaneous resolution during crystallization.

## 1. Chemical context

Compounds with a 2,3-diaryl-2,3,5,6-tetra­hydro-4*H*-1,3-thia­zin-4-one scaffold have been shown to have a variety of bioactivities, including anti­parasite (Malfara *et al.*, 2021 ▸), anti­tumor (Chen *et al.*, 2012 ▸; Dandia *et al.*, 2013 ▸), anti­fungal (Ten Haken & Beatrice, 1983 ▸; Qu *et al.*, 2013 ▸.; Dandia *et al.*, 2004 ▸; Krumkains, 1984 ▸), anti­tubercular (Dandia *et al.*, 2004 ▸), anti­diabetic (Arya *et al.*, 2012 ▸), inhibition of cannabinoid receptor 1 (CB1) (Choi *et al.*, 2008 ▸), inhibition of angiogenesis (possible treatment of eye disease, neoplasm, arteriosclerosis, arthritis, psoriasis, diabetes, and mellitus) (Yi *et al.*, 2012 ▸), regulation of plant growth (Krumkains, 1984 ▸), anti­microbial (Mogilaiah *et al.*, 1999 ▸) and anti­bacterial (Mahdi & Rasheed, 2023 ▸) effects.

We have previously used our T3P method to synthesize two series of these compounds in which the aryl rings on positions 2 and 3 had a variety of substituents on them (Silverberg *et al.*, 2020 ▸, 2025 ▸). Currently, another series in which one of the aryl rings is a heteroaryl is being synthesized. Here we report the syntheses and crystal structures of two new compounds in this series, one in which there is a thia­zole ring attached to N3 (compound **1**) and one in which there is a furan ring on C2 (compound **2**). Thia­zole and furan derivatives are each known for their biological activity (Niu *et al.*, 2023 ▸; Nivrutti, 2024 ▸) and could have inter­esting effects on the activity of the 2,3,5,6-tetra­hydro-4*H*-1,3-thia­zin-4-ones.
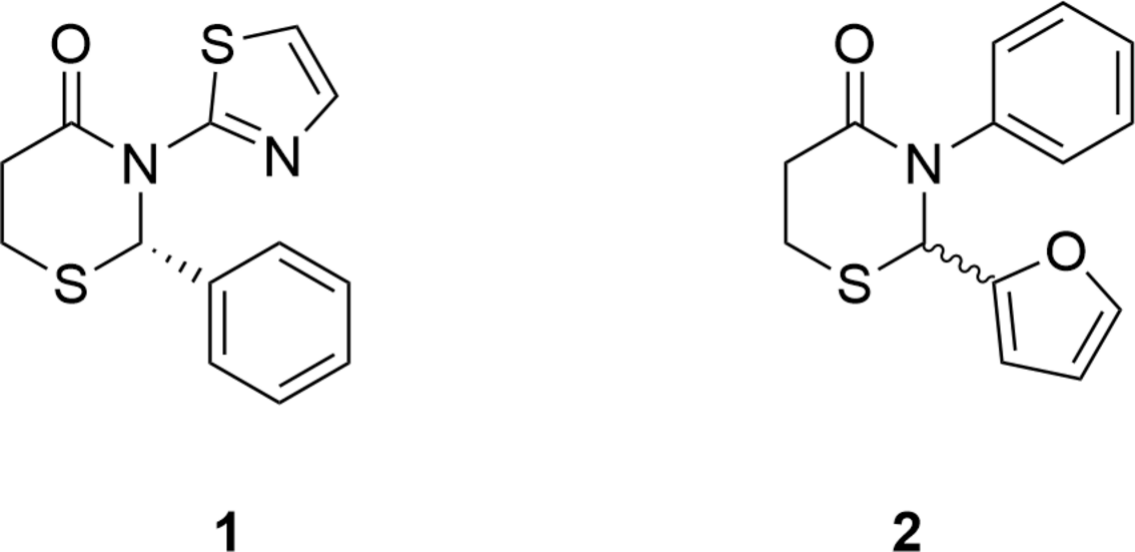


The spontaneous resolution of a racemic solution by direct crystallization to form a conglomerate, a mechanical mixture of separate homochiral crystals, is an uncommon but well-known phenomenon, recognized first by Pasteur (Pasteur, 1848 ▸; Jacques *et al.*, 1981 ▸; Eliel & Wilen, 1994 ▸; Pérez-García & Amabilino, 2007 ▸). It has even been used in the production of chiral active pharmaceutical ingredients (Bredikhin & Bredikhina, 2017 ▸). However, the reasons why this occurs with a minority of mol­ecules are not well understood (Pérez-García & Amabilino, 2007 ▸) and have not yet yielded to attempts to predict occurrence (D’Oria *et al.*, 2010 ▸; Pérez-García & Amabilino, 2007 ▸).

## 2. Structural commentary

**Figure 1:**
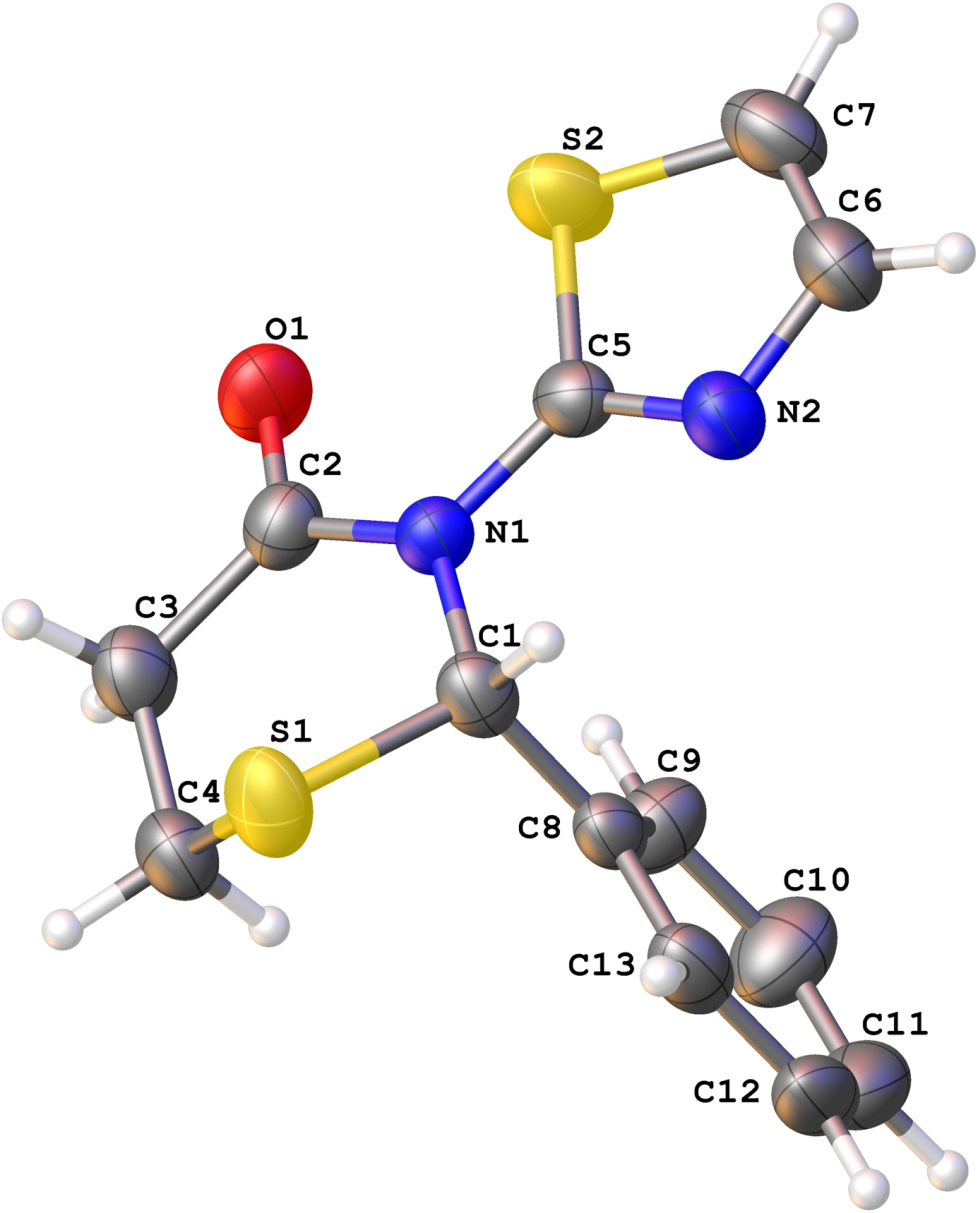
The mol­ecular structure of **1** with displacement ellipsoids drawn at the 50% probability level.

**Figure 2:**
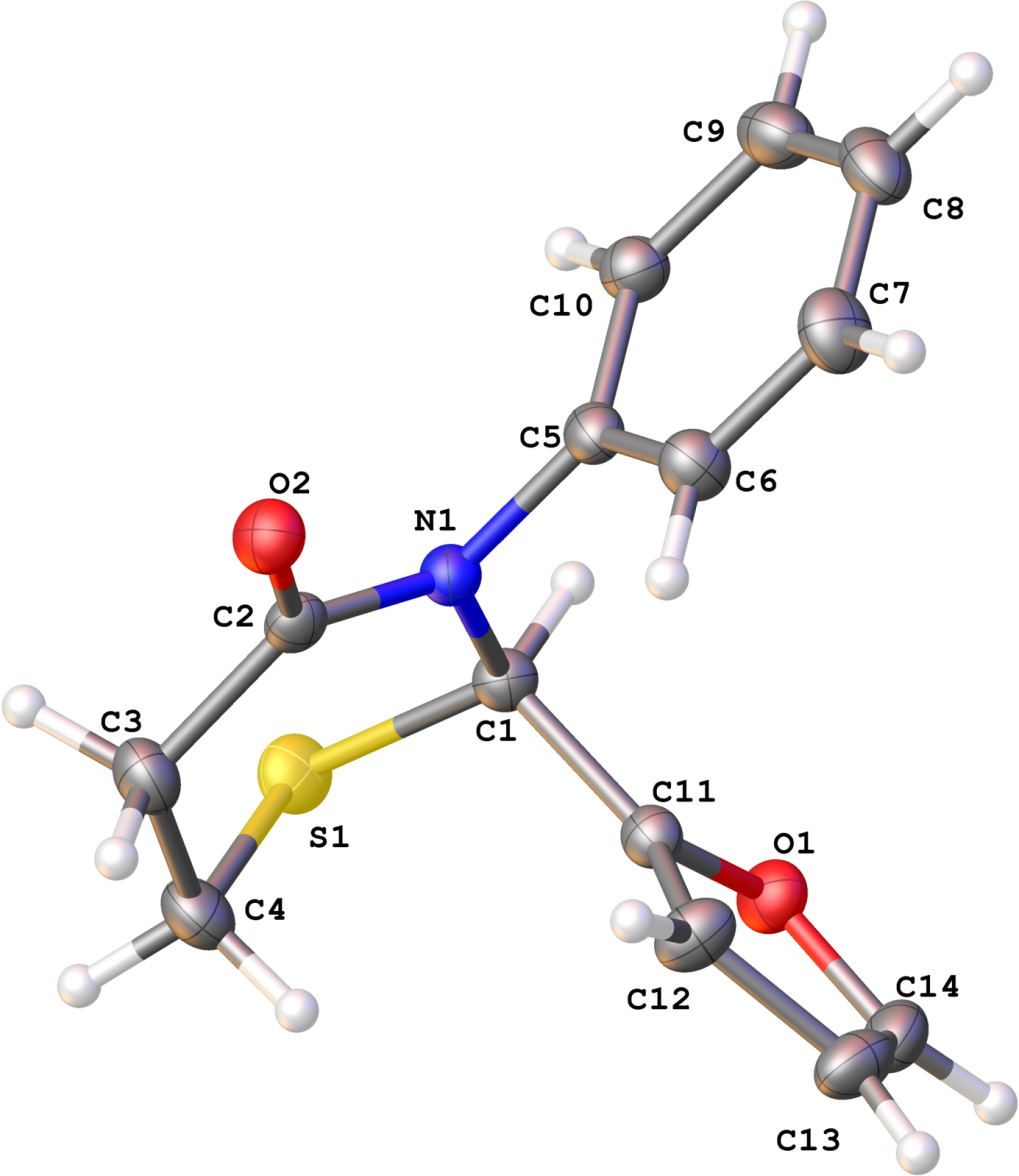
The mol­ecular structure of **2** with displacement ellipsoids drawn at the 50% probability level.

Compounds **1** (Fig. 1 ▸) and **2** (Fig. 2 ▸) crystallize in the ortho­rhom­bic *P*2_1_2_1_2_1_ and monoclinic *P*2_1_/*c* space groups, respectively, with a single mol­ecule in the respective asymmetric units. The chosen crystal of **1** was found to be composed of mol­ecules with an *S* configuration at the stereogenic atom C1 [Flack parameter 0.013 (9)]. We had previously observed a similar spontaneous resolution in another thia­zine compound (Yennawar, Bradley *et al.*, 2018 ▸). The crystal of **2** belongs to a centrosymmetric space group and must be a racemic mixture of mol­ecules.

The thia­zine rings of **1** and **2** both display a half-chair (pucker) conformation [*Q* = 0.694 (2), 0.6482 (11) Å; θ = 47.75 (17), 47.02 (10)°; φ = 347.4 (3), 349.01 (16)°, respectively] where the sulfur atom forms the back of the chair. We have observed this mode of puckering earlier (Yennawar, Yang *et al.*, 2016 ▸; Yennawar, Fox *et al.*, 2016 ▸; Yennawar, Bradley *et al.*, 2018 ▸), which is different from the screw-boat (Yennawar, Bendinsky *et al.*, 2014 ▸; Yennawar, Fox *et al.*, 2017 ▸; Yennawar, Noble *et al.*, 2017 ▸; Yennawar, Mal *et al.*, 2023 ▸) conformation or the envelope pucker (Yennawar, Singh *et al.*, 2014 ▸).

In the structure of **1**, the C5/C6/C7/N2/S2 thia­zole ring and the five non-S atoms of the thia­zine ring are almost coplanar, while the dihedral angle between the thia­zole and the C8–C13 phenyl ring is 81.91 (13)°. In **2**, the C5–C10 phenyl and C11–C14/O1 furan rings flip positions as compared to the phenyl and thia­zole rings in **1.** The phenyl ring is *gauche* with respect to the plane of the thia­zine ring’s five non-S atoms. The dihedral angle between the phenyl and furan ring planes is 88.45 (7)°.

## 3. Supra­molecular features

**Figure 3:**
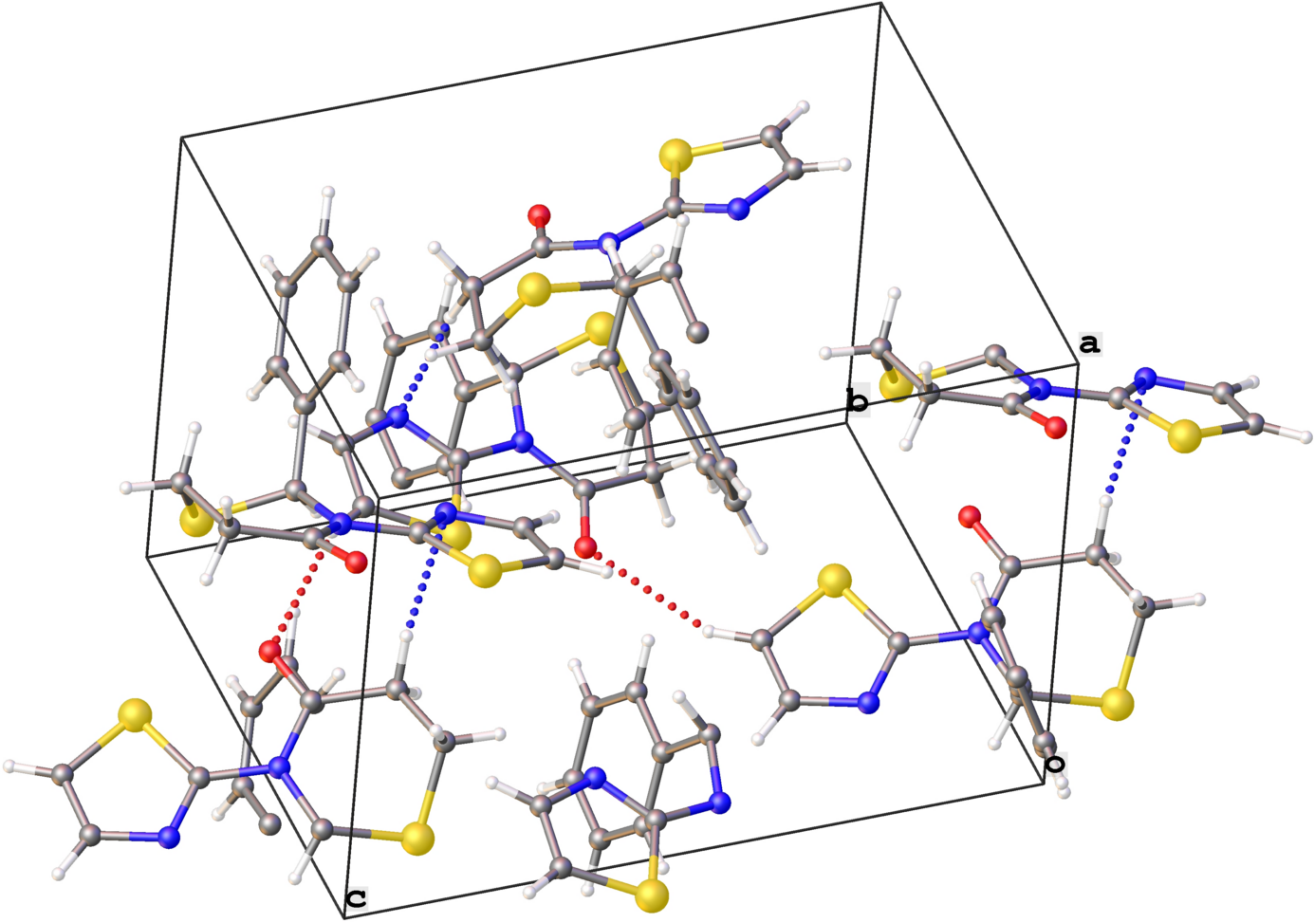
Crystal packing diagram of **1** with red dotted lines for C—H⋯O contacts and blue dotted lines for the C—H⋯N contacts.

**Figure 4:**
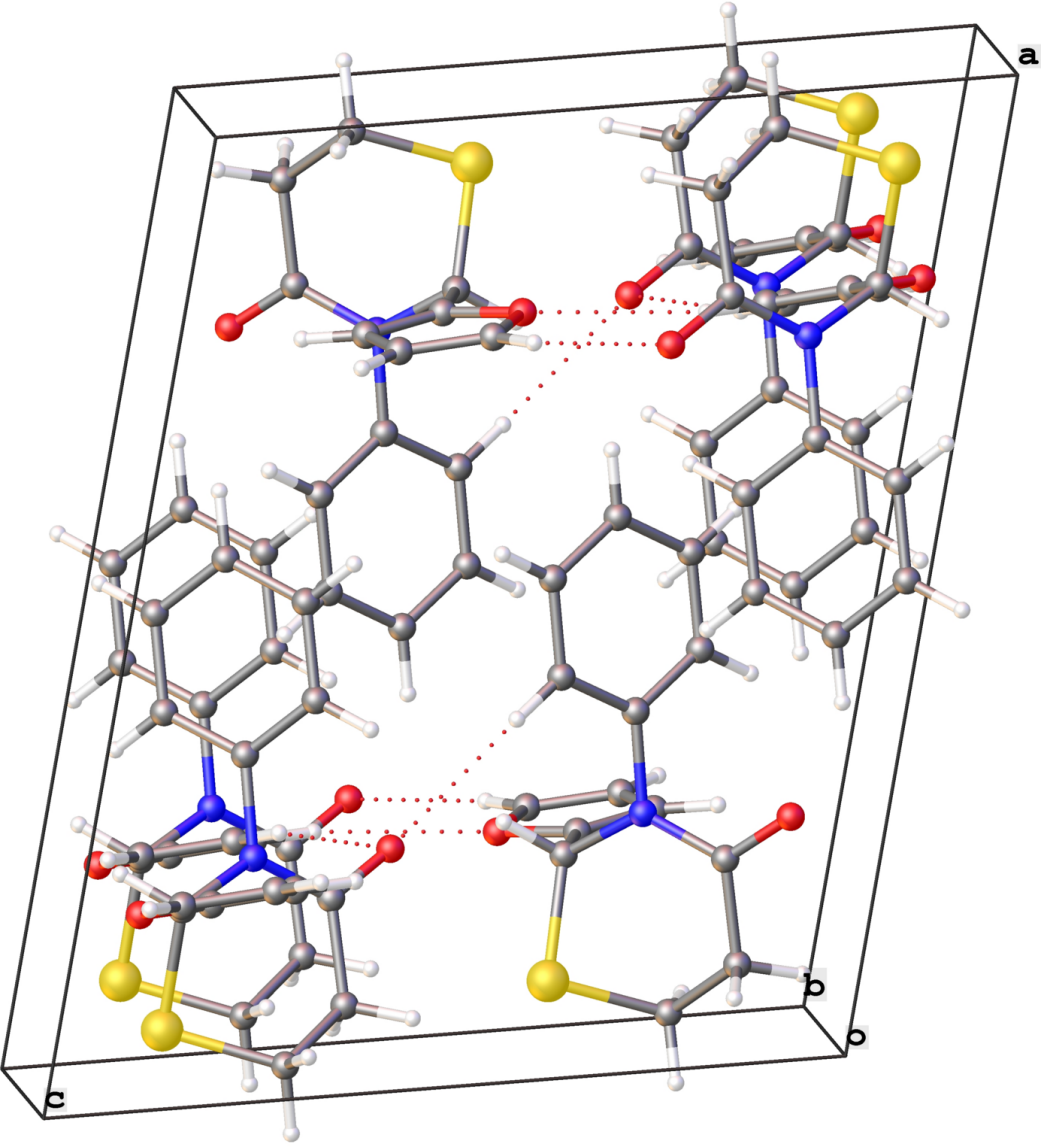
Crystal packing diagram of **2** showing alternating layers of amphiphilic and hydro­phobic regions. Red dotted lines represent the C—H⋯O contacts.

The extended structure of **1** (Table 1 ▸, Fig. 3 ▸) features C7—H7⋯O1 hydrogen bonds forming mol­ecular chains along the *c*-axis direction and C3—H3B⋯N2 hydrogen bonds forming chains along the *a*-axis direction. No π–π stacking inter­actions or layering of any kind is observed in the extended structure. The crystal of **2** on the other hand (Fig. 4 ▸) is brought about by a continuous and extensive network of inter­molecular hydrogen bonds (Table 2 ▸). These include the oxygen atom on the substituted thia­zine ring acting as a double acceptor for the C13—H13⋯O2 and C14—H14⋯O2 bonds and also between symmetry-related furan rings (C12—H12⋯O1). These inter­actions result in infinite layers of furan and thia­zine moieties lying parallel to the (100) plane. Additional inter­action between a carbon atom of the phenyl ring and the oxygen atom on the thia­zine ring (C10—H10⋯O2) and the π–π stacking of the phenyl rings between pairs of mol­ecules further consolidate the packing. Alternating layers of amphiphilic (furan and thia­zine) and hydro­phobic (phen­yl) entities, parallel to the (100) plane are a feature of this structure.

## 4. Database survey

We have previously reported the crystal structures of 2,3-diphenyl-2,3,5,6-tetra­hydro-4*H*-1,3-thia­zin-4-one (Yennawar & Silverberg, 2014 ▸, 2015 ▸), *N*-[(2*S*,5*R*)-4-oxo-2,3-diphenyl-1,3-thia­zinan-5-yl] acetamide 0.375 hydrate (Yennawar *et al.*, 2015 ▸), (2*S*)-2-(3-nitro­phen­yl)-3-phenyl-2,3,5,6-tetra­hydro-4*H*-1,3-thia­zin-4-one (Yennawar, Bradley *et al.*, 2018 ▸), *rac*-2-(4-nitro­phen­yl)-3-phenyl-2,3,5,6-tetra­hydro-4*H*-1,3-thia­zin-4-one (Yennawar, Bradley *et al.*, 2018 ▸), racemic (R*,R*)-2,2′-(1,4-phenyl­ene)bis­(3-phenyl-2,3,5,6-tetra­hydro-4*H*-1,3-thia­zin-4-one) (Yennawar *et al.*, 2021 ▸), and *meso*-3,3′-(1,4-phenyl­ene)bis­(2-phenyl-2,3,5,6-tetra­hydro-4*H*-1,3-thia­zin-4-one) (Yennawar, Moyer & Silverberg, 2018 ▸). A literature survey did not reveal crystal structures of any other 2,3-diaryl-2,3,5,6-tetra­hydro-4*H*-1,3-thia­zin-4-ones.

## 5. Synthesis and crystallization

TLC plates (silica gel GF, 250-micron, 10 × 20 cm, cat. No. P21521) were purchased from Miles Scientific. TLCs were visualized under short wave UV, and then with I_2_, and then by spraying with ceric ammonium nitrate/sulfuric acid and heating. Infrared spectra were run on a Thermo-Fisher NICOLET iS50 FT-IR using a diamond-ATR attachment for direct powder analysis (Penn State Schuylkill). ^1^H and ^13^C NMR experiments (Penn State’s shared NMR facility, University Park) were carried out on a Bruker Avance-III-HD 500.20-MHz (1H frequency) instrument using a 5 mm Prodigy (liquid nitro­gen cooled) BBOBB-1H/19F/D Z-GRD cryoprobe. Samples were dissolved in pyridine-*d*5 and analyzed at RT. Typical conditions for ^1^H acquisition were 1 s relaxation delay, the acquisition time of 3.28 s, the spectral width of 10 kHz, 32 scans. Spectra were zero-filled to 128k points, and multiplied by exponential multiplication (EM with LB = 0.3 Hz) prior to FT. For ^13^C experiments, data were acquired with power-gated ^1^H decoupling using a 2 s relaxation delay, with an acquisition time of 1.1 s, spectral width of 29.8 kHz, and 256 scans. Spectra were zero-filled once, and multiplied by EM with LB = 2 Hz prior to FT. MS samples were analyzed for accurate mass by LCMS on a SCIEX Exion LC with a SCIEX 5600+ TripleTOF MS. Separation was achieved on an Agilent Infinity LabPoroshell column 120 EC-C18, 2.1 X 50mm, 2.7-micron particle (p/n 699775-902), column maintained at 313 K. Elution using a reversed phase gradient of 100% (water with 0.1% formic acid) ramped to 100% (aceto­nitrile with 0.1% formic acid) over 10 min at a flowrate of 0.4 mL min^−1^. The MS was scanned over 50–1200 Da and calibrated with the SCIEX APCI positive calibrant solution (Part 4460131) prior to sample analysis. Samples were analyzed in ESI positive mode with a DP = 100 V, CE = 10, GAS1 = GAS2 = 60 psi, curtain = 30 psi, ISV = 5500 V, and source temperature of 773 K (Villanova University). Melting points were performed on a Vernier Melt Station (Penn State Schuylkill). Suitable crystals were selected and sequentially mounted using a nylon loop and a dab of paratone oil.

**2-Phenyl-3-(thia­zol-2-yl)-2,3,5,6-tetra­hydro-4*****H*****-1,3-thia­zin-4-one, 1**: A two-necked 25 ml round-bottom flask was oven-dried, cooled under N_2_, and charged with a stir bar. 2-Amino­thia­zole (0.6005 g, 6.00 mmol) and benzaldehyde (0.6369 g, 6.00 mmol) were added. 2-Methyl­tetra­hydro­furan (2.3 ml) was added and the solution was stirred for five minutes. 3-Mercaptopropionic acid (0.6379 g, 6.00 mmol) was added followed by pyridine (2.9 ml, 36 mmol). Finally, 2,4,6-tripropyl-1,3,5,2,4,6-trioxatri­phospho­rinane-2,4,6-trioxide (T3P) in 2-methyl­tetra­hydro­furan (50 weight percent; 11 ml, 18 mmol) was added. The reaction was stirred at room temperature and followed by TLC, then poured into a separatory funnel with di­chloro­methane (20 ml). The mixture was washed with water (10 ml). The aqueous was then extracted twice with di­chloro­methane (10 ml each). The organics were combined and washed with saturated sodium bicarbonate (10 ml) and then saturated sodium chloride (10 ml). The organic extract was dried over sodium sulfate and concentrated under vacuum to give a crude mixture. After chromatography on 30 g silica gel with mixtures of ethyl acetate and hexa­nes (gradient from 30% ethyl acetate to 70%), recrystallization from methanol solution gave an off-white solid (0.1186 g, 7% yield). m.p.: 426–427 K. Colorless blocks of **1** for crystallography were grown by slow evaporation from methanol solution. ^1^H NMR (*d*5-pyridine) δ 7.65 (*s*, 1H), 7.47 (*d*, *J* = 3.7 Hz, 1H), 7.39 (*d*, *J* = 7.8 Hz, 2H), 7.26 (*t*, *J* = 7.6 Hz, 2H), 7.21 (*d*, *J* = 7.2 Hz, 1H), 7.12 (*d*, *J* = 3.7 Hz, 1H), 3.16–2.93 (*m*, 2H), 2.83–2.73 (*m*, 1H), 2.65–2.55 (*m*, 1H). ^13^C NMR (d5-pyridine) δ 168.5, 158.4, 140.1, 137.4, 128.7, 127.8, 126.4, 115.9, 61.7, 34.4, 20.8. HRMS (*m*/*z*): [*M* + H^+^] of 277.0465 is consistent with calculated [M + H]^+^ of 277.0464. IR (neat, cm^−1^): 1647 (C=O).

**2-(Furan-2-yl)-3-phenyl-2,3,5,6-tetra­hydro-4*****H*****-1,3-thia­zin-4-one, 2**: A two-necked 25 ml round-bottom flask was oven-dried, cooled under N_2_, and charged with a stir bar. Aniline (0.5585 g, 6.00 mmol) and furfural (0.5764 g, 6.00 mmol) were added. 2-Methyl­tetra­hydro­furan (2.3 ml) was added and the solution was stirred for five minutes. Pyridine (2.9 ml, 36 mmol) and 2,4,6-tripropyl-1,3,5,2,4,6-trioxatri­phospho­rinane-2,4,6-trioxide (T3P) in 2-methyl­tetra­hydro­furan (50 weight percent; 11 ml, 18 mmol) were added. Lastly, 3-mercaptopropionic acid (0.52 ml, 6.00 mmol) was added dropwise. The reaction was stirred at room temperature and followed by TLC, then poured into a separatory funnel with ethyl acetate (20 ml). The mixture was washed with water (10 ml). The aqueous layer was then extracted twice with ethyl acetate (10 ml each). The organics were combined and washed with saturated sodium bicarbonate (10 ml) and then saturated sodium chloride (10 ml). The organic extract was dried over sodium sulfate and concentrated under vacuum to give a crude mixture. After chromatography on 30 g silica gel with mixtures of ethyl acetate and hexa­nes (gradient from 30% ethyl acetate to 70%), recrystallization from ethanol gave a light orange solid (0.538 g, 35% yield). m.p.: 380.1–380.9 K (decomposition). Yellow blocks of **2** were grown by slow evaporation from ethanol solution. ^1^H NMR (*d*5-pyridine) δ 7.43 (d, *J* = 8.2 Hz, 2H), 7.30 (*t*, *J* = 7.9 Hz, 2H), 7.18 (*d*, *J* = 14.9 Hz, 2H), 6.49 (*d*, *J* = 3.4 Hz, 1H), 6.38 (*dd*, *J* = 3.3, 1.8 Hz, 1H), 6.32 (*s*, 1H), 3.23–3.13 (*m*, 1H), 3.07–2.92 (*m*, 2H), 2.88–2.79 (*m*, 1H). ^13^C NMR (*d*5-pyridine) δ 168.7, 152.1, 143.2, 143.1, 129.1, 127.6, 127.2, 110.8, 109.1, 59.7, 34.7, 23.3. HRMS (*m*/*z*): [*M* + H^+^] of 260.0737 is consistent with calculated [*M* + H]^+^ of 260.0739. IR (neat, cm^−1^): 1623 (C=O).

## 6. Refinement

Crystal data, data collection and structure refinement details are summarized in Table 3 ▸. The H atoms were placed geometrically and allowed to ride on their parent C atoms during refinement, with C—H distances of 0.95 Å (aromatic), 0.97 Å (meth­ylene) and 1.0 Å (methyne) and with *U*_iso_(H) = 1.2*U*_eq_ (aromatic or methyl­ene C) or 1.5*U*_eq_ (methyl C).

## Supplementary Material

Crystal structure: contains datablock(s) 1, 2. DOI: 10.1107/S2056989025009193/hb8168sup1.cif

Structure factors: contains datablock(s) 1. DOI: 10.1107/S2056989025009193/hb81681sup2.hkl

Supporting information file. DOI: 10.1107/S2056989025009193/hb81681sup4.mol

Structure factors: contains datablock(s) 2. DOI: 10.1107/S2056989025009193/hb81682sup3.hkl

Supporting information file. DOI: 10.1107/S2056989025009193/hb81682sup5.mol

Supporting information file. DOI: 10.1107/S2056989025009193/hb81681sup6.cml

Supporting information file. DOI: 10.1107/S2056989025009193/hb81682sup7.cml

CCDC references: 2496686, 2496685

Additional supporting information:  crystallographic information; 3D view; checkCIF report

## Figures and Tables

**Table 1:** Hydrogen-bond geometry (Å, °) for **1**[Chem scheme1]

*D*—H⋯*A*	*D*—H	H⋯*A*	*D*⋯*A*	*D*—H⋯*A*
C3—H3*B*⋯N2^i^	0.99	2.53	3.503 (4)	169
C7—H7⋯O1^ii^	0.95	2.62	3.495 (3)	154

Symmetry codes: (i) 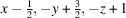; (ii) 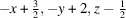.

**Table 2:** Hydrogen-bond geometry (Å, °) for **2**[Chem scheme1]

*D*—H⋯*A*	*D*—H	H⋯*A*	*D*⋯*A*	*D*—H⋯*A*
C10—H10⋯O2^i^	0.95	2.54	3.4276 (16)	155
C12—H12⋯O1^ii^	0.95	2.58	3.4583 (16)	153
C14—H14⋯O2^iii^	0.95	2.50	3.4252 (16)	166
C13—H13⋯O2^iv^	0.95	2.43	3.3641 (16)	168

Symmetry codes: (i) 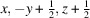; (ii) 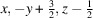; (iii) 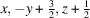; (iv) 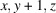.

**Table 3:** Experimental details

	**1**	**2**
Crystal data		
Chemical formula	C_13_H_12_N_2_OS_2_	C_14_H_13_NO_2_S
*M*_r_	276.37	259.31
Crystal system, space group	Orthorhombic, *P*2_1_2_1_2_1_	Monoclinic, *P*2_1_/*c*
Temperature (K)	173	173
*a*, *b*, *c* (Å)	9.71506 (15), 10.04091 (13), 13.06169 (18)	14.0188 (2), 8.0422 (1), 11.3223 (2)
α, β, γ (°)	90, 90, 90	90, 104.066 (1), 90
*V* (Å^3^)	1274.14 (3)	1238.22 (3)
*Z*	4	4
Radiation type	Cu *K*α	Cu *K*α
μ (mm^−1^)	3.69	2.27
Crystal size (mm)	0.15 × 0.10 × 0.05	0.19 × 0.18 × 0.09

Data collection		
Diffractometer	ROD, SynergyCustom system, HyPix-Arc 150	ROD, SynergyCustom system, HyPix-Arc 150
Absorption correction	Multi-scan (*CrysAlis PRO*; Rigaku OD, 2025 ▸)	Multi-scan (*CrysAlis PRO*; Rigaku OD, 2025 ▸)
*T*_min_, *T*_max_	0.820, 1.000	0.813, 1.000
No. of measured, independent and observed [*I* > 2σ(*I*)] reflections	15582, 2577, 2456	7838, 2435, 2322
*R*_int_	0.037	0.021
(sin θ/λ)_max_ (Å^−1^)	0.631	0.630

Refinement		
*R*[*F*^2^ > 2σ(*F*^2^)], *wR*(*F*^2^), *S*	0.028, 0.073, 1.05	0.029, 0.080, 1.08
No. of reflections	2577	2435
No. of parameters	164	164
H-atom treatment	H-atom parameters constrained	H-atom parameters constrained
Δρ_max_, Δρ_min_ (e Å^−3^)	0.14, −0.18	0.28, −0.30
Absolute structure	Flack *x* determined using 979 quotients [(*I*^+^)−(*I*^−^)]/[(*I*^+^)+(*I*^−^)] (Parsons *et al.*, 2013 ▸)	–
Absolute structure parameter	0.013 (9)	–

Computer programs: *CrysAlis PRO* (Rigaku OD, 2025), *SHELXT* (Sheldrick, 2015*a* ▸), *SHELXL2018/3* (Sheldrick, 2015*b* ▸) and *OLEX2* (Dolomanov *et al.*, 2009 ▸).

## supplementary crystallographic information

### (2S)-2-Phenyl-3-(thiazol-2-yl)-2,3,5,6-tetrahydro-4H-1,3-thiazin-4-one (1) Crystal data

**Table:**

C_13_H_12_N_2_OS_2_	*D*_x_ = 1.441 Mg m^−^^3^
*M**_r_* = 276.37	Cu *K*α radiation, λ = 1.54184 Å
Orthorhombic, *P*2_1_2_1_2_1_	Cell parameters from 8550 reflections
*a* = 9.71506 (15) Å	θ = 3.4–73.4°
*b* = 10.04091 (13) Å	µ = 3.69 mm^−^^1^
*c* = 13.06169 (18) Å	*T* = 173 K
*V* = 1274.14 (3) Å^3^	Block, colorless
*Z* = 4	0.15 × 0.10 × 0.05 mm
*F*(000) = 576	

### (2S)-2-Phenyl-3-(thiazol-2-yl)-2,3,5,6-tetrahydro-4H-1,3-thiazin-4-one (1) Data collection

**Table:**

ROD, SynergyCustom system, HyPix-Arc 150 diffractometer	2577 independent reflections
Radiation source: Rotating-anode X-ray tube, Rigaku (Cu) X-ray Source	2456 reflections with *I* > 2σ(*I*)
Mirror monochromator	*R*_int_ = 0.037
Detector resolution: 10.0000 pixels mm^-1^	θ_max_ = 76.5°, θ_min_ = 5.6°
ω scans	*h* = −10→12
Absorption correction: multi-scan (CrysAlisPro; Rigaku OD, 2025)	*k* = −12→12
*T*_min_ = 0.820, *T*_max_ = 1.000	*l* = −16→16
15582 measured reflections	

### (2S)-2-Phenyl-3-(thiazol-2-yl)-2,3,5,6-tetrahydro-4H-1,3-thiazin-4-one (1) Refinement

**Table:**

Refinement on *F*^2^	H-atom parameters constrained
Least-squares matrix: full	*w* = 1/[σ^2^(*F*_o_^2^) + (0.0356*P*)^2^ + 0.1897*P*] where *P* = (*F*_o_^2^ + 2*F*_c_^2^)/3
*R*[*F*^2^ > 2σ(*F*^2^)] = 0.028	(Δ/σ)_max_ < 0.001
*wR*(*F*^2^) = 0.073	Δρ_max_ = 0.14 e Å^−^^3^
*S* = 1.05	Δρ_min_ = −0.18 e Å^−^^3^
2577 reflections	Extinction correction: SHELXL-2018/3 (Sheldrick 2015b), Fc^*^=kFc[1+0.001xFc^2^λ^3^/sin(2θ)]^-1/4^
164 parameters	Extinction coefficient: 0.0019 (4)
0 restraints	Absolute structure: Flack *x* determined using 979 quotients [(*I*^+^)-(*I*^-^)]/[(*I*^+^)+(*I*^-^)] (Parsons *et al.*, 2013)
Hydrogen site location: inferred from neighbouring sites	Absolute structure parameter: 0.013 (9)

### (2S)-2-Phenyl-3-(thiazol-2-yl)-2,3,5,6-tetrahydro-4H-1,3-thiazin-4-one (1) Special details

**Table:**

Geometry. All esds (except the esd in the dihedral angle between two l.s. planes) are estimated using the full covariance matrix. The cell esds are taken into account individually in the estimation of esds in distances, angles and torsion angles; correlations between esds in cell parameters are only used when they are defined by crystal symmetry. An approximate (isotropic) treatment of cell esds is used for estimating esds involving l.s. planes.

### (2S)-2-Phenyl-3-(thiazol-2-yl)-2,3,5,6-tetrahydro-4H-1,3-thiazin-4-one (1) Fractional atomic coordinates and isotropic or equivalent isotropic displacement parameters (Å^2^)

**Table:**

	*x*	*y*	*z*	*U*_iso_*/*U*_eq_	
S1	0.89569 (8)	0.55653 (7)	0.61560 (5)	0.0551 (2)	
S2	0.80954 (8)	0.91269 (7)	0.30808 (6)	0.0584 (2)	
O1	0.7233 (2)	0.91808 (17)	0.49817 (15)	0.0560 (5)	
N1	0.8157 (2)	0.71837 (18)	0.45942 (14)	0.0372 (4)	
N2	0.8863 (2)	0.6695 (2)	0.29155 (16)	0.0492 (5)	
C1	0.8492 (2)	0.5775 (2)	0.48283 (18)	0.0403 (5)	
H1	0.932918	0.554839	0.441811	0.048*	
C8	0.7369 (3)	0.4831 (2)	0.44846 (17)	0.0394 (5)	
C2	0.7542 (2)	0.8056 (2)	0.52664 (19)	0.0410 (5)	
C9	0.6083 (3)	0.5254 (3)	0.4168 (2)	0.0533 (7)	
H9	0.586998	0.617694	0.417852	0.064*	
C5	0.8384 (2)	0.7550 (2)	0.35675 (18)	0.0381 (5)	
C6	0.9008 (3)	0.7284 (3)	0.1967 (2)	0.0548 (7)	
H6	0.933586	0.680501	0.138953	0.066*	
C13	0.7641 (4)	0.3466 (3)	0.4476 (2)	0.0536 (7)	
H13	0.851329	0.314590	0.469296	0.064*	
C4	0.7364 (3)	0.6209 (3)	0.6645 (2)	0.0511 (7)	
H4A	0.657947	0.571637	0.634444	0.061*	
H4B	0.733136	0.610383	0.739804	0.061*	
C3	0.7273 (3)	0.7666 (3)	0.6364 (2)	0.0488 (6)	
H3A	0.793298	0.815724	0.680090	0.059*	
H3B	0.633938	0.798117	0.654775	0.059*	
C12	0.6630 (5)	0.2569 (3)	0.4149 (2)	0.0687 (10)	
H12	0.681842	0.164056	0.415077	0.082*	
C7	0.8660 (3)	0.8566 (3)	0.1911 (2)	0.0600 (8)	
H7	0.871548	0.909530	0.130911	0.072*	
C10	0.5096 (3)	0.4347 (3)	0.3836 (3)	0.0681 (9)	
H10	0.422186	0.465784	0.361365	0.082*	
C11	0.5374 (4)	0.3017 (3)	0.3825 (3)	0.0682 (9)	
H11	0.469717	0.240301	0.359493	0.082*	

### (2S)-2-Phenyl-3-(thiazol-2-yl)-2,3,5,6-tetrahydro-4H-1,3-thiazin-4-one (1) Atomic displacement parameters (Å^2^)

**Table:**

	*U*^11^	*U*^22^	*U*^33^	*U*^12^	*U*^13^	*U*^23^
S1	0.0709 (4)	0.0553 (4)	0.0391 (3)	0.0174 (3)	−0.0116 (3)	0.0017 (3)
S2	0.0820 (5)	0.0416 (4)	0.0515 (4)	0.0087 (3)	0.0069 (4)	0.0116 (3)
O1	0.0800 (13)	0.0353 (9)	0.0527 (10)	0.0115 (9)	0.0006 (9)	−0.0027 (8)
N1	0.0463 (10)	0.0299 (9)	0.0354 (10)	0.0011 (8)	0.0000 (9)	−0.0002 (8)
N2	0.0680 (14)	0.0432 (12)	0.0364 (11)	−0.0006 (11)	0.0043 (10)	−0.0001 (9)
C1	0.0505 (13)	0.0361 (12)	0.0343 (11)	0.0096 (10)	0.0023 (9)	0.0018 (10)
C8	0.0581 (14)	0.0317 (11)	0.0284 (10)	0.0009 (10)	0.0081 (10)	−0.0006 (9)
C2	0.0458 (12)	0.0363 (12)	0.0409 (12)	0.0014 (10)	−0.0022 (10)	−0.0055 (10)
C9	0.0532 (15)	0.0405 (13)	0.0660 (18)	−0.0014 (12)	0.0024 (13)	−0.0090 (12)
C5	0.0424 (11)	0.0350 (11)	0.0369 (12)	−0.0034 (9)	−0.0027 (9)	0.0022 (9)
C6	0.0694 (17)	0.0591 (17)	0.0359 (13)	−0.0055 (14)	0.0043 (13)	0.0028 (12)
C13	0.088 (2)	0.0378 (13)	0.0352 (13)	0.0093 (13)	0.0069 (13)	0.0027 (10)
C4	0.0691 (17)	0.0500 (15)	0.0341 (12)	−0.0018 (13)	0.0029 (12)	0.0007 (11)
C3	0.0583 (15)	0.0479 (14)	0.0401 (13)	0.0051 (12)	0.0069 (11)	−0.0044 (11)
C12	0.131 (3)	0.0312 (14)	0.0434 (15)	−0.0125 (17)	0.0188 (18)	−0.0030 (11)
C7	0.0734 (19)	0.0614 (18)	0.0452 (15)	−0.0007 (14)	0.0053 (14)	0.0158 (14)
C10	0.0595 (17)	0.0628 (19)	0.082 (2)	−0.0120 (15)	0.0055 (16)	−0.0157 (19)
C11	0.089 (2)	0.0566 (18)	0.0594 (18)	−0.0269 (17)	0.0190 (18)	−0.0084 (17)

### (2S)-2-Phenyl-3-(thiazol-2-yl)-2,3,5,6-tetrahydro-4H-1,3-thiazin-4-one (1) Geometric parameters (Å, º)

**Table:**

S1—C1	1.804 (2)	C9—C10	1.392 (4)
S1—C4	1.795 (3)	C6—H6	0.9500
S2—C5	1.730 (2)	C6—C7	1.333 (4)
S2—C7	1.718 (3)	C13—H13	0.9500
O1—C2	1.226 (3)	C13—C12	1.400 (5)
N1—C1	1.484 (3)	C4—H4A	0.9900
N1—C2	1.377 (3)	C4—H4B	0.9900
N1—C5	1.408 (3)	C4—C3	1.510 (4)
N2—C5	1.295 (3)	C3—H3A	0.9900
N2—C6	1.380 (3)	C3—H3B	0.9900
C1—H1	1.0000	C12—H12	0.9500
C1—C8	1.513 (3)	C12—C11	1.368 (5)
C8—C9	1.383 (4)	C7—H7	0.9500
C8—C13	1.395 (3)	C10—H10	0.9500
C2—C3	1.510 (4)	C10—C11	1.363 (5)
C9—H9	0.9500	C11—H11	0.9500
			
C4—S1—C1	94.83 (12)	C8—C13—H13	119.9
C7—S2—C5	88.58 (13)	C8—C13—C12	120.1 (3)
C2—N1—C1	124.80 (19)	C12—C13—H13	119.9
C2—N1—C5	120.63 (19)	S1—C4—H4A	110.0
C5—N1—C1	114.25 (18)	S1—C4—H4B	110.0
C5—N2—C6	110.1 (2)	H4A—C4—H4B	108.4
S1—C1—H1	106.6	C3—C4—S1	108.26 (19)
N1—C1—S1	111.35 (16)	C3—C4—H4A	110.0
N1—C1—H1	106.6	C3—C4—H4B	110.0
N1—C1—C8	112.22 (19)	C2—C3—C4	118.2 (2)
C8—C1—S1	113.11 (16)	C2—C3—H3A	107.8
C8—C1—H1	106.6	C2—C3—H3B	107.8
C9—C8—C1	123.2 (2)	C4—C3—H3A	107.8
C9—C8—C13	118.0 (3)	C4—C3—H3B	107.8
C13—C8—C1	118.8 (3)	H3A—C3—H3B	107.1
O1—C2—N1	119.9 (2)	C13—C12—H12	119.7
O1—C2—C3	119.0 (2)	C11—C12—C13	120.6 (3)
N1—C2—C3	121.0 (2)	C11—C12—H12	119.7
C8—C9—H9	119.5	S2—C7—H7	124.8
C8—C9—C10	121.0 (3)	C6—C7—S2	110.4 (2)
C10—C9—H9	119.5	C6—C7—H7	124.8
N1—C5—S2	124.30 (17)	C9—C10—H10	119.7
N2—C5—S2	115.03 (17)	C11—C10—C9	120.5 (3)
N2—C5—N1	120.7 (2)	C11—C10—H10	119.7
N2—C6—H6	122.0	C12—C11—H11	120.1
C7—C6—N2	116.0 (3)	C10—C11—C12	119.7 (3)
C7—C6—H6	122.0	C10—C11—H11	120.1
			
S1—C1—C8—C9	115.7 (2)	C2—N1—C5—S2	6.7 (3)
S1—C1—C8—C13	−64.8 (3)	C2—N1—C5—N2	−174.5 (2)
S1—C4—C3—C2	49.7 (3)	C9—C8—C13—C12	0.4 (4)
O1—C2—C3—C4	166.8 (3)	C9—C10—C11—C12	0.2 (5)
N1—C1—C8—C9	−11.3 (3)	C5—S2—C7—C6	0.1 (2)
N1—C1—C8—C13	168.2 (2)	C5—N1—C1—S1	153.07 (17)
N1—C2—C3—C4	−14.8 (4)	C5—N1—C1—C8	−79.0 (2)
N2—C6—C7—S2	−0.5 (4)	C5—N1—C2—O1	−2.7 (4)
C1—S1—C4—C3	−65.9 (2)	C5—N1—C2—C3	179.0 (2)
C1—N1—C2—O1	−175.7 (2)	C5—N2—C6—C7	0.8 (4)
C1—N1—C2—C3	5.9 (4)	C6—N2—C5—S2	−0.7 (3)
C1—N1—C5—S2	−179.52 (17)	C6—N2—C5—N1	−179.5 (2)
C1—N1—C5—N2	−0.8 (3)	C13—C8—C9—C10	−1.0 (4)
C1—C8—C9—C10	178.4 (3)	C13—C12—C11—C10	−0.9 (5)
C1—C8—C13—C12	−179.1 (2)	C4—S1—C1—N1	58.12 (18)
C8—C9—C10—C11	0.7 (5)	C4—S1—C1—C8	−69.34 (18)
C8—C13—C12—C11	0.6 (4)	C7—S2—C5—N1	179.1 (2)
C2—N1—C1—S1	−33.5 (3)	C7—S2—C5—N2	0.3 (2)
C2—N1—C1—C8	94.5 (3)		

### (2S)-2-Phenyl-3-(thiazol-2-yl)-2,3,5,6-tetrahydro-4H-1,3-thiazin-4-one (1) Hydrogen-bond geometry (Å, º)

**Table:**

*D*—H···*A*	*D*—H	H···*A*	*D*···*A*	*D*—H···*A*
C3—H3*B*···N2^i^	0.99	2.53	3.503 (4)	169
C7—H7···O1^ii^	0.95	2.62	3.495 (3)	154

Symmetry codes: (i) *x*−1/2, −*y*+3/2, −*z*+1; (ii) −*x*+3/2, −*y*+2, *z*−1/2.

### 2-(Furan-2-yl)-3-phenyl-2,3,5,6-tetrahydro-4H-1,3-thiazin-4-one (2) Crystal data

**Table:**

C_14_H_13_NO_2_S	*F*(000) = 544
*M**_r_* = 259.31	*D*_x_ = 1.391 Mg m^−^^3^
Monoclinic, *P*2_1_/*c*	Cu *K*α radiation, λ = 1.54184 Å
*a* = 14.0188 (2) Å	Cell parameters from 5692 reflections
*b* = 8.0422 (1) Å	θ = 3.2–76.2°
*c* = 11.3223 (2) Å	µ = 2.27 mm^−^^1^
β = 104.066 (1)°	*T* = 173 K
*V* = 1238.22 (3) Å^3^	Block, yellow
*Z* = 4	0.19 × 0.18 × 0.09 mm

### 2-(Furan-2-yl)-3-phenyl-2,3,5,6-tetrahydro-4H-1,3-thiazin-4-one (2) Data collection

**Table:**

ROD, SynergyCustom system, HyPix-Arc 150 diffractometer	2322 reflections with *I* > 2σ(*I*)
Detector resolution: 10.0000 pixels mm^-1^	*R*_int_ = 0.021
ω scans	θ_max_ = 76.3°, θ_min_ = 3.3°
Absorption correction: multi-scan (CrysAlisPro; Rigaku OD, 2025)	*h* = −17→15
*T*_min_ = 0.813, *T*_max_ = 1.000	*k* = −9→9
7838 measured reflections	*l* = −12→13
2435 independent reflections	

### 2-(Furan-2-yl)-3-phenyl-2,3,5,6-tetrahydro-4H-1,3-thiazin-4-one (2) Refinement

**Table:**

Refinement on *F*^2^	Hydrogen site location: inferred from neighbouring sites
Least-squares matrix: full	H-atom parameters constrained
*R*[*F*^2^ > 2σ(*F*^2^)] = 0.029	*w* = 1/[σ^2^(*F*_o_^2^) + (0.0403*P*)^2^ + 0.4195*P*] where *P* = (*F*_o_^2^ + 2*F*_c_^2^)/3
*wR*(*F*^2^) = 0.080	(Δ/σ)_max_ < 0.001
*S* = 1.08	Δρ_max_ = 0.28 e Å^−^^3^
2435 reflections	Δρ_min_ = −0.30 e Å^−^^3^
164 parameters	Extinction correction: SHELXL-2018/3 (Sheldrick 2015b), Fc^*^=kFc[1+0.001xFc^2^λ^3^/sin(2θ)]^-1/4^
0 restraints	Extinction coefficient: 0.0077 (5)

### 2-(Furan-2-yl)-3-phenyl-2,3,5,6-tetrahydro-4H-1,3-thiazin-4-one (2) Special details

**Table:**

Geometry. All esds (except the esd in the dihedral angle between two l.s. planes) are estimated using the full covariance matrix. The cell esds are taken into account individually in the estimation of esds in distances, angles and torsion angles; correlations between esds in cell parameters are only used when they are defined by crystal symmetry. An approximate (isotropic) treatment of cell esds is used for estimating esds involving l.s. planes.

### 2-(Furan-2-yl)-3-phenyl-2,3,5,6-tetrahydro-4H-1,3-thiazin-4-one (2) Fractional atomic coordinates and isotropic or equivalent isotropic displacement parameters (Å^2^)

**Table:**

	*x*	*y*	*z*	*U*_iso_*/*U*_eq_	
S1	0.07806 (2)	0.43110 (4)	0.36542 (3)	0.02744 (13)	
O2	0.23770 (7)	0.25789 (11)	0.10605 (8)	0.0256 (2)	
O1	0.21764 (7)	0.77273 (11)	0.44109 (8)	0.0254 (2)	
N1	0.24650 (7)	0.38603 (13)	0.28567 (9)	0.0190 (2)	
C11	0.21704 (8)	0.66614 (15)	0.34668 (10)	0.0194 (3)	
C5	0.35291 (8)	0.37345 (15)	0.31819 (11)	0.0202 (3)	
C4	0.19546 (9)	0.32966 (15)	0.17485 (11)	0.0200 (3)	
C1	0.20443 (8)	0.48549 (15)	0.37027 (10)	0.0196 (3)	
H1	0.244178	0.461383	0.454540	0.024*	
C10	0.39909 (9)	0.29220 (16)	0.42481 (11)	0.0240 (3)	
H10	0.361323	0.242939	0.474755	0.029*	
C12	0.22949 (10)	0.75125 (16)	0.24929 (12)	0.0272 (3)	
H12	0.231244	0.706306	0.172252	0.033*	
C6	0.40772 (9)	0.44727 (16)	0.24559 (12)	0.0254 (3)	
H6	0.375659	0.503820	0.173134	0.030*	
C14	0.23214 (10)	0.92929 (16)	0.39920 (13)	0.0288 (3)	
H14	0.236273	1.028377	0.445845	0.035*	
C3	0.08435 (9)	0.34646 (18)	0.13502 (12)	0.0280 (3)	
H3A	0.067243	0.381193	0.048541	0.034*	
H3B	0.055757	0.234554	0.138570	0.034*	
C9	0.50141 (10)	0.28352 (17)	0.45798 (12)	0.0293 (3)	
H9	0.533662	0.227828	0.530735	0.035*	
C8	0.55618 (9)	0.35590 (18)	0.38510 (13)	0.0310 (3)	
H8	0.625913	0.349154	0.407707	0.037*	
C7	0.50956 (10)	0.43809 (18)	0.27934 (13)	0.0305 (3)	
H7	0.547376	0.488261	0.229822	0.037*	
C2	0.03348 (9)	0.46475 (18)	0.20431 (12)	0.0297 (3)	
H2A	−0.038455	0.446094	0.180119	0.036*	
H2B	0.046518	0.581040	0.184200	0.036*	
C13	0.23958 (10)	0.92243 (16)	0.28411 (14)	0.0299 (3)	
H13	0.249607	1.013374	0.234946	0.036*	

### 2-(Furan-2-yl)-3-phenyl-2,3,5,6-tetrahydro-4H-1,3-thiazin-4-one (2) Atomic displacement parameters (Å^2^)

**Table:**

	*U*^11^	*U*^22^	*U*^33^	*U*^12^	*U*^13^	*U*^23^
S1	0.02374 (18)	0.0304 (2)	0.0316 (2)	−0.00259 (12)	0.01330 (13)	0.00038 (12)
O2	0.0287 (5)	0.0262 (5)	0.0225 (5)	0.0000 (4)	0.0073 (3)	−0.0041 (4)
O1	0.0306 (5)	0.0235 (5)	0.0217 (5)	−0.0001 (4)	0.0056 (3)	−0.0048 (3)
N1	0.0185 (5)	0.0189 (5)	0.0200 (5)	0.0004 (4)	0.0053 (4)	−0.0012 (4)
C11	0.0191 (5)	0.0204 (6)	0.0188 (6)	0.0008 (4)	0.0045 (4)	−0.0031 (4)
C5	0.0195 (6)	0.0183 (6)	0.0223 (6)	0.0006 (4)	0.0042 (4)	−0.0036 (5)
C4	0.0233 (6)	0.0162 (5)	0.0200 (6)	−0.0008 (4)	0.0047 (5)	0.0027 (4)
C1	0.0205 (5)	0.0206 (6)	0.0186 (6)	0.0005 (5)	0.0066 (4)	0.0004 (5)
C10	0.0254 (6)	0.0225 (6)	0.0238 (6)	0.0012 (5)	0.0053 (5)	0.0000 (5)
C12	0.0364 (7)	0.0220 (6)	0.0263 (7)	0.0014 (5)	0.0140 (5)	0.0011 (5)
C6	0.0243 (6)	0.0275 (7)	0.0245 (6)	−0.0001 (5)	0.0063 (5)	0.0015 (5)
C14	0.0271 (7)	0.0198 (6)	0.0389 (8)	−0.0013 (5)	0.0068 (6)	−0.0056 (5)
C3	0.0218 (6)	0.0325 (7)	0.0272 (7)	−0.0019 (5)	0.0013 (5)	−0.0023 (5)
C9	0.0279 (7)	0.0290 (7)	0.0273 (7)	0.0057 (5)	−0.0004 (5)	−0.0023 (5)
C8	0.0193 (6)	0.0355 (8)	0.0363 (7)	0.0025 (5)	0.0031 (5)	−0.0088 (6)
C7	0.0237 (6)	0.0367 (8)	0.0332 (7)	−0.0033 (5)	0.0110 (5)	−0.0034 (6)
C2	0.0189 (6)	0.0346 (7)	0.0335 (7)	0.0017 (5)	0.0023 (5)	0.0002 (6)
C13	0.0314 (7)	0.0198 (6)	0.0415 (8)	0.0014 (5)	0.0148 (6)	0.0041 (5)

### 2-(Furan-2-yl)-3-phenyl-2,3,5,6-tetrahydro-4H-1,3-thiazin-4-one (2) Geometric parameters (Å, º)

**Table:**

S1—C1	1.8125 (12)	C12—C13	1.4295 (18)
S1—C2	1.7992 (14)	C6—H6	0.9500
O2—C4	1.2308 (15)	C6—C7	1.3874 (18)
O1—C11	1.3687 (14)	C14—H14	0.9500
O1—C14	1.3779 (16)	C14—C13	1.333 (2)
N1—C5	1.4505 (15)	C3—H3A	0.9900
N1—C4	1.3621 (15)	C3—H3B	0.9900
N1—C1	1.4765 (15)	C3—C2	1.5169 (19)
C11—C1	1.4954 (17)	C9—H9	0.9500
C11—C12	1.3448 (18)	C9—C8	1.385 (2)
C5—C10	1.3870 (17)	C8—H8	0.9500
C5—C6	1.3876 (18)	C8—C7	1.385 (2)
C4—C3	1.5188 (16)	C7—H7	0.9500
C1—H1	1.0000	C2—H2A	0.9900
C10—H10	0.9500	C2—H2B	0.9900
C10—C9	1.3935 (18)	C13—H13	0.9500
C12—H12	0.9500		
			
C2—S1—C1	95.20 (6)	C7—C6—H6	120.2
C11—O1—C14	106.07 (10)	O1—C14—H14	124.7
C5—N1—C1	114.93 (9)	C13—C14—O1	110.60 (11)
C4—N1—C5	119.22 (10)	C13—C14—H14	124.7
C4—N1—C1	125.08 (10)	C4—C3—H3A	107.7
O1—C11—C1	116.58 (10)	C4—C3—H3B	107.7
C12—C11—O1	110.14 (11)	H3A—C3—H3B	107.1
C12—C11—C1	133.28 (11)	C2—C3—C4	118.65 (11)
C10—C5—N1	119.18 (11)	C2—C3—H3A	107.7
C10—C5—C6	120.58 (11)	C2—C3—H3B	107.7
C6—C5—N1	120.21 (11)	C10—C9—H9	119.9
O2—C4—N1	120.88 (11)	C8—C9—C10	120.13 (12)
O2—C4—C3	118.05 (11)	C8—C9—H9	119.9
N1—C4—C3	121.01 (11)	C9—C8—H8	119.9
S1—C1—H1	107.2	C9—C8—C7	120.17 (12)
N1—C1—S1	113.03 (8)	C7—C8—H8	119.9
N1—C1—C11	109.10 (9)	C6—C7—H7	120.0
N1—C1—H1	107.2	C8—C7—C6	120.09 (13)
C11—C1—S1	112.71 (8)	C8—C7—H7	120.0
C11—C1—H1	107.2	S1—C2—H2A	109.7
C5—C10—H10	120.3	S1—C2—H2B	109.7
C5—C10—C9	119.36 (12)	C3—C2—S1	109.81 (9)
C9—C10—H10	120.3	C3—C2—H2A	109.7
C11—C12—H12	126.6	C3—C2—H2B	109.7
C11—C12—C13	106.73 (12)	H2A—C2—H2B	108.2
C13—C12—H12	126.6	C12—C13—H13	126.8
C5—C6—H6	120.2	C14—C13—C12	106.46 (12)
C5—C6—C7	119.67 (12)	C14—C13—H13	126.8
			
O2—C4—C3—C2	−167.11 (12)	C4—N1—C1—C11	−92.17 (13)
O1—C11—C1—S1	77.57 (11)	C4—C3—C2—S1	−47.73 (15)
O1—C11—C1—N1	−156.00 (9)	C1—S1—C2—C3	61.81 (10)
O1—C11—C12—C13	0.57 (15)	C1—N1—C5—C10	64.62 (14)
O1—C14—C13—C12	−0.11 (16)	C1—N1—C5—C6	−113.03 (12)
N1—C5—C10—C9	−178.37 (11)	C1—N1—C4—O2	174.61 (11)
N1—C5—C6—C7	178.32 (11)	C1—N1—C4—C3	−8.21 (17)
N1—C4—C3—C2	15.63 (18)	C1—C11—C12—C13	−178.65 (13)
C11—O1—C14—C13	0.45 (14)	C10—C5—C6—C7	0.71 (19)
C11—C12—C13—C14	−0.28 (16)	C10—C9—C8—C7	0.4 (2)
C5—N1—C4—O2	5.23 (17)	C12—C11—C1—S1	−103.25 (15)
C5—N1—C4—C3	−177.59 (11)	C12—C11—C1—N1	23.17 (18)
C5—N1—C1—S1	−156.14 (8)	C6—C5—C10—C9	−0.74 (19)
C5—N1—C1—C11	77.62 (12)	C14—O1—C11—C1	178.73 (10)
C5—C10—C9—C8	0.2 (2)	C14—O1—C11—C12	−0.63 (13)
C5—C6—C7—C8	−0.1 (2)	C9—C8—C7—C6	−0.4 (2)
C4—N1—C5—C10	−124.95 (12)	C2—S1—C1—N1	−55.69 (9)
C4—N1—C5—C6	57.40 (16)	C2—S1—C1—C11	68.60 (9)
C4—N1—C1—S1	34.08 (14)		

### 2-(Furan-2-yl)-3-phenyl-2,3,5,6-tetrahydro-4H-1,3-thiazin-4-one (2) Hydrogen-bond geometry (Å, º)

**Table:**

*D*—H···*A*	*D*—H	H···*A*	*D*···*A*	*D*—H···*A*
C10—H10···O2^i^	0.95	2.54	3.4276 (16)	155
C12—H12···O1^ii^	0.95	2.58	3.4583 (16)	153
C14—H14···O2^iii^	0.95	2.50	3.4252 (16)	166
C13—H13···O2^iv^	0.95	2.43	3.3641 (16)	168

Symmetry codes: (i) *x*, −*y*+1/2, *z*+1/2; (ii) *x*, −*y*+3/2, *z*−1/2; (iii) *x*, −*y*+3/2, *z*+1/2; (iv) *x*, *y*+1, *z*.
